# Genomic and biological aspects of resistance to selective poly(ADP‐ribose) glycohydrolase inhibitor PDD00017273 in human colorectal cancer cells

**DOI:** 10.1002/cnr2.1709

**Published:** 2022-08-26

**Authors:** Kaede Tsuda, Chinatsu Kurasaka, Yoko Ogino, Akira Sato

**Affiliations:** ^1^ Department of Biochemistry and Molecular Biology, Faculty of Pharmaceutical Sciences Tokyo University of Science Chiba Japan; ^2^ Department of Gene Regulation, Faculty of Pharmaceutical Sciences Tokyo University of Science Chiba Japan

**Keywords:** drug resistance, exome sequencing, PARG inhibitor, PDD00017273, poly(ADP‐ribose) glycohydrolase

## Abstract

**Background:**

Poly(ADP‐ribose) glycohydrolase (PARG) is a key enzyme in poly(ADP‐ribose) (PAR) metabolism and a potential anticancer target. Many drug candidates have been developed to inhibit its enzymatic activity. Additionally, PDD00017273 is an effective and selective inhibitor of PARG at the first cellular level.

**Aims:**

Using human colorectal cancer (CRC) HCT116 cells, we investigated the molecular mechanisms and tumor biological aspects of the resistance to PDD00017273.

**Methods and results:**

HCT116R^PDD^, a variant of the human CRC cell line HCT116, exhibits resistance to the PARG inhibitor PDD00017273. HCT116R^PDD^ cells contained specific mutations of *PARG* and *PARP1*, namely, *PARG* mutation Glu352Gln and *PARP1* mutation Lys134Asn, as revealed by exome sequencing. Notably, the levels of PARG protein were comparable between HCT116R^PDD^ and HCT116. In contrast, the PARP1 protein levels in HCT116R^PDD^ were significantly lower than those in HCT116. Consequently, the levels of intracellular poly(ADP‐ribosyl)ation were elevated in HCT116R^PDD^ compared to HCT116. Interestingly, HCT116R^PDD^ cells did not exhibit cross‐resistance to COH34, an additional PARG inhibitor.

**Conclusion:**

Our findings suggest that the mutated PARG acquires PDD00017273 resistance due to structural modifications. In addition, our findings indicate that PDD00017273 resistance induces mutation and *PARP* downregulation. These discoveries collectively provide a better understanding of the anticancer candidate PARG inhibitors in terms of resistance mechanisms and anticancer strategies.

## 1 INTRODUCTION

Poly (ADP‐ribosyl)ation (PARylation) is a reversible posttranslational modification mediated by poly(ADP‐ribose) polymerases (PARPs) and resolved primarily by poly(ADP‐ribose) glycohydrolase (PARG).^1^, ^2^, ^3^, ^4^, ^5^ Poly (ADP‐ribose) (PAR) is degraded to ADP‐ribose by PARG and ADP‐ribosyl hydrolase (ARH3) after synthesis by PARPs.^6^, ^7^ PARylation regulated‐enzymes, namely, PARP and PARG, are desirable targets for the development of anticancer drugs.^1^, ^2^, ^5^ In fact, Olaparib is the first‐line drug used clinically to inhibit PARP.^5^, ^8^ In contrast, although numerous natural and synthetic compounds have been reported as potential PARG inhibitors, their selectivity, activity, and cell permeability still present obstacles.^4^, ^9^, ^10^ The PARG inhibitor PDD00017273 inhibits PARG efficiently and selectively with a biochemical IC_50_ = 26 nM while being devoid of activity against PARP1 and ARH3.^11^ In addition, PDD00017273 is the first effective inhibitor of cellular permeability and activity at the cellular level; however, it is clinically unsuitable because of its limited bioavailability.^5^, ^11^ However, this PARG inhibitor offers experimental and fundamental insights into its function of PARG and its significance as a cancer treatment molecular target. Recently, Chen and Yu reported the specific and potent PARG inhibitor COH34.^12^ This novel PARG inhibitor inhibits tumor cell growth in cell‐based and xenograft mouse cancer models.^12^ These PARG inhibitors, PDD00017273 and COH34, have been studied and discussed in relation to *BRCA1/2* mutations, also known as the synthetic lethal genes for PARP inhibitors, and their anticancer activity.^11^, ^12^

PARG exhibits oncogenic activity and is considered a potential therapeutic target for numerous tumors. Previous studies demonstrated that PARG silencers and PARG inhibitors, such as PDD00017273, inhibit tumor cell growth and increase radiosensitization in the MCF‐7 breast cancer cell line.^13^, ^14^, ^15^ Higher PARG expression is associated with a poor prognosis in breast cancer, particularly in the HER2‐positive and triple‐negative subtypes.^16^ In contrast, PARG depletion significantly inhibits cell growth and metastasis of triple‐negative breast tumors.^16^ As previously reported, PARG deficiency causes PAR accumulation and DNA repair delay.^17^, ^18^ PARG dysfunction increases the sensitivity of human pancreatic ductal adenocarcinoma (PDAC) MIAPaCa2 cells and human colorectal cancer RKO cells^17^ to alkylating agents. In vitro and in vivo experiments showed that silencing PARG inhibited PDAC tumor growth.^19^, ^20^ Additionally, PARG inhibitors inhibited cell survival in numerous PDAC cell lines.^20^ Multiple studies have identified *BRCA1/2*, *PALB2*, *FAM175A*, *BARD1*, *TIMELESS*, *HUS1*, *RFC2*, *DUSP22*, and *POLB* as candidate synthetic lethal genes associated with PARG.^5^, ^11^, ^12^, ^13^, ^14^, ^21^, ^22^, ^23^

Several studies have previously investigated the mechanisms of resistance to anticancer PARP inhibitors and demonstrated the association between reversion mutations, epigenetic modification, restoration of PARylation, and pharmacological modification of the resistance machinery.^24^, ^25^ However, the resistance of cancer to PARG inhibitors and its resistance mechanism remains unclear. We believe that research on the mechanism of PARG inhibitor resistance is essential for comprehending the role of PARG in cancer and its anticancer drug target. This investigation established a PARG inhibitor (PDD00017273)‐resistant HCT116 cell line (HCT116R^PDD^) and evaluated its tumor biological characteristics. Exome sequencing revealed the status of genetic alterations between parental HCT116 and HCT116R^PDD^ cells. In addition, the anticancer sensitivity of HCT116 and HCT116R^PDD^ cells to the novel PARG inhibitor COH34 was evaluated. Furthermore, potential cellular resistance mechanisms to the PARG inhibitor PDD00017273 in identified gene mutations and their regulatory gene expression were discussed.

## 2 METHODS

### 2.1 Reagents

PDD00017273 and COH34, two selective PARG inhibitors, were purchased from MedChemExpress (Monmouth Junction, New Jersey, United States). PDD00017273 was maintained at −25°C as a 0.1 M stock solution in dimethyl sulfoxide (DMSO, Sigma–Aldrich; Merck KGaA, Darmstadt, Germany). COH34 was kept as a 0.05 M stock solution in DMSO at −25°C. In the experiment, these stock solutions were used with up to one freeze–thaw cycle.

### 2.2 Cell culture

The human colon cancer cell line HCT116 was obtained from the American Type Culture Collection. The parental and PDD00017273‐resistant HCT116 cell lines were cultured in DMEM medium with 10% heat‐inactivated fetal bovine serum, 100 units/ml penicillin, and 100‐μg/ml streptomycin in a 37°C incubator under an atmosphere with 5% CO_2_ and 100% relative humidity. Based on trypan blue dye exclusion, cell viability was estimated with a hematocytometer.

### 2.3 Generation of the PARG inhibitor‐resistant HCT116 cell line

HCT116 cells resistant to PARG inhibitors were generated by exposing cells to 10 μM PDD00017273 for ~1 month followed by 30 μM PDD00017273 for ~1 month. A derivative of HCT116, designated HCT116R^PDD^, was isolated. HCT116R^PDD^ cells were cultured in the presence of 30 μM PDD00017273.

### 2.4 Cell activity WST‐8 assay

As previously described, cell viability tests were conducted.^26^, ^27^ The WST‐8 (Cell Counting Kit‐8) cell proliferation assay determined cell viability (Dojindo, Tokyo, Japan). Briefly, cells were seeded into 96‐well plates (1000 cells per well) in triplicate and then treated with various anticancer drug concentrations or DMSO (as a negative control). After 72 h of incubation, WST‐8 reagent was added to each well, and the plate was placed in a 5% CO_2_ incubator at 37°C for one additional h. At 450 nm, absorbance was measured using a Tecan microplate reader (Mannedorf, Switzerland). The EC_50_ value was defined as the drug concentration that inhibited cell proliferation by 50%. The EC_50_ values represent the mean ± SD of three independent experiments.

### 2.5 Colony formation assay

A colony formation test was conducted as described previously.^26^, ^27^, ^28^ HCT116 and HCT116R^PDD^ cells were dissociated with Accutase, suspended in media, inoculated into 6‐well plates (200 cells per well) in duplicate, and then incubated overnight. As a negative control, the cells were treated with DMSO. Cells were treated with various concentrations of drugs. After incubation for 10 days, the cells were fixed in 4% formaldehyde solution and stained using 0.1% (wt/vol) crystal violet, and the number of colonies in each well was determined. The EC_50_ values are the mean ± SD of three independent experiments.

### 2.6 Exome sequencing analysis

DNA extraction was performed as described previously.^26^, ^28^ Following the manufacturer's instructions, genomic DNA was extracted from using a DNeasy Blood & Tissue Kit (QIAGEN, Venlo, Netherlands). Following the Agilent SureSelectXT Low Input Target Enrichment protocol for an Illumina paired‐end sequencing library, exome capture libraries were constructed. The DNA quantity and quality were determined using PicoGreen (Thermo Fisher Scientific, Waltham, MA, United States) and agarose gel electrophoresis. The fragments of adapter‐modified genomic DNA were amplified by PCR. The final purified product was quantified using TapeStation DNA screentape D1000 (Agilent, Santa Clara, CA, United States). SureSelect Human All Exon V6 was used to perform target enrichment (Agilent). Then, paired‐end sequencing of 150 bp reads was performed using the Illumina NovaSeq 6000 sequencing system (Illumina, San Diego, CA, USA). Integrale, Co., Ltd. (Tokyo, Japan) and Macrogen Global Headquarters (Seoul, South Korea) performed exome sequencing on the parental HCT116 and HCT116R^PDD^ cells. In HCT116 cells, the average throughput depth of the target region (X) was 188.3, while in HCT116R^PDD^ cells, it was 118.3. The target region was covered 99.7% of the time in HCT116 cells and 99.6% of the time in HCT116R^PDD^ cells.

### 2.7 Western blot analysis

Western blot analysis was performed as previously described^26^, ^27^, ^28^, ^29^ with the following antibodies: mouse anti‐PARG (D8B10) monoclonal antibody (1:500; MABS61, Sigma‐Aldrich), rabbit anti‐PARP polyclonal antibody (1:1000; #9542, Cell Signaling Technologies, Danvers, MA, United States), mouse anti‐BRCA1(D‐9) monoclonal antibody (1:200; sc‐6954, Santa Cruz Biotechnology, Dallas, MA, United States), mouse anti‐poly(ADP‐ribose) monoclonal antibody (10H) (1:500; ALX‐804‐220, Enzo Life Sciences, Farmingdale, NY, United States), rabbit anti‐GAPDH antibody (1:20000; 2275‐PC‐100, Trevigen, Gaithersburg, MD, United States), horseradish peroxidase‐linked anti‐rabbit IgG (1:20000, GE Healthcare, Marlborough, MA, United States), and horseradish peroxidase‐linked whole antibody anti‐mouse IgG (1:20000, GE Healthcare).

### 2.8 Statistical analysis

Statistical analyses were conducted using GraphPad Prism 9 software. The data were presented as the mean ± SE. Student's *t* tests and one‐way analysis of variance (ANOVA) were used to evaluate significant differences between groups (ANOVA). *p < .05 and **p < .01 were considered statistically significant.

## 3 RESULTS

### 3.1 Establishment of PDD00017273‐resistant HCT116 cells

**FIGURE 1:**
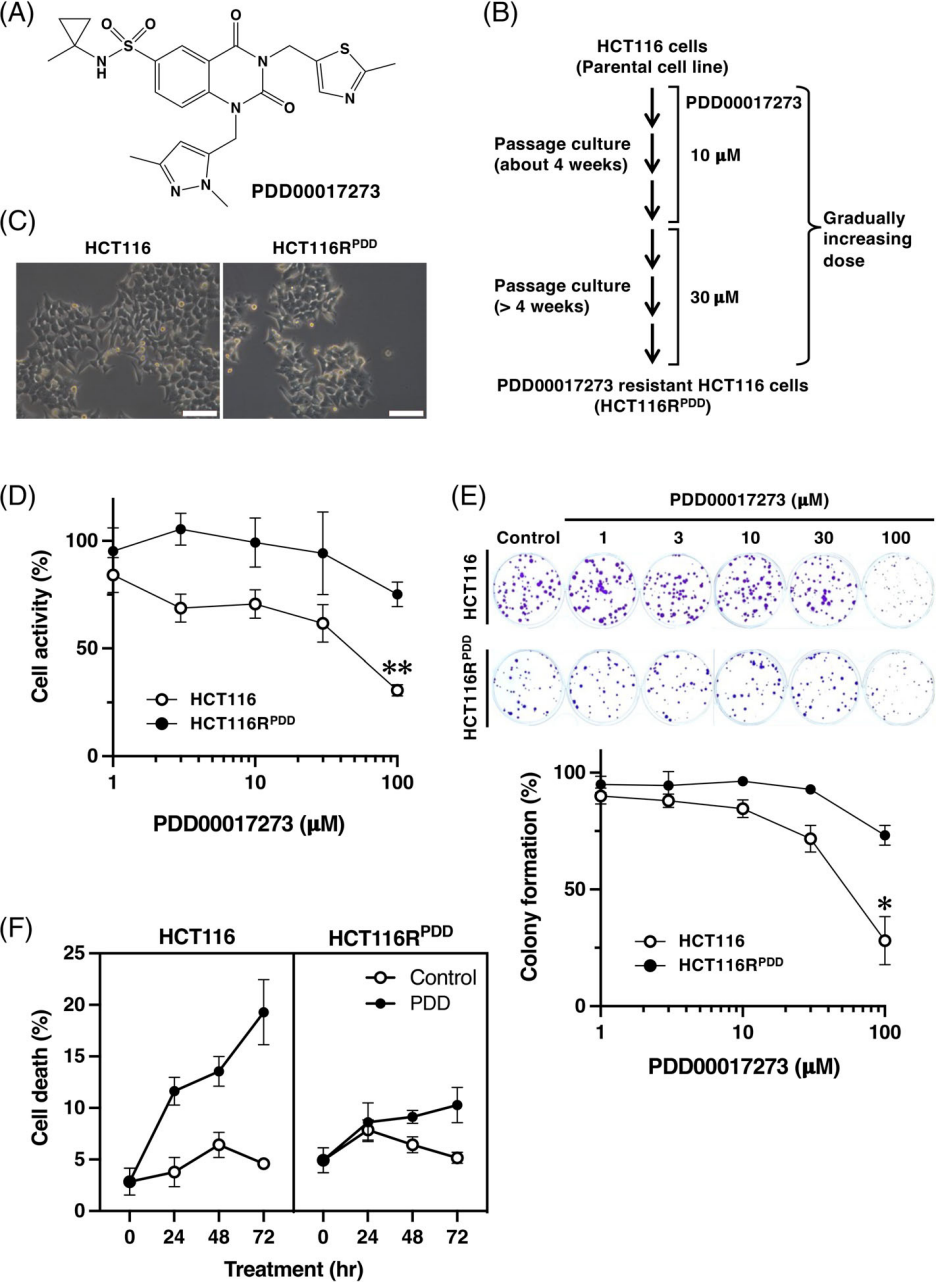
Generation of PARG inhibitor PDD00017273‐resistant HCT116 cells. (A) The chemical composition of PDD00017273. (B) Diagrammatic depiction of the development of PDD00017273‐resistant HCT116 cells (HCT116R^PDD^). (C) The morphological features of the cells were analyzed using a Leica DMi1 microscope with LAS V4.12 and a 20× objective. Scale bar = 100 μm. (D) After 72 h of treatment with PDD00017273, the activity of HCT116 and HCT116R^PDD^ cells was evaluated. The results were the average of three independent samples, with error bars displaying ±SE. White circle represents HCT116 cells; black circle represents HCT116R^PDD^ cells. **p < .01 (HCT116 cells versus HCT116R^PDD^ cells treated with 100 μM PDD00017273). (E) The colony formation assay was used to assess the drug sensitivity of HCT116 and HCT116R^PDD^. The HCT116 and HCT116R^PDD^ cells were treated with the indicated concentration of PDD00017273 and incubated for 10 days. Colony formation (%) represents the average of three independent experiments, and each conducted in duplicate, with error bars showing ±SE of the triplicate average. The white circle represents HCT116 cells; black circle represents HCT116R^PDD^ cells. *p < .05 (HCT116 cells versus HCT116R^PDD^ cells treated with 100 μM PDD00017273). (F) Cell death (%) of the HCT116 and HCT116R^PDD^ cells treated with 100 μM PDD00017273. The white circle, Control indicates solvent (DMSO) alone; black circle, PDD indicates PDD00017273, *p* = .0768 (analysis of variance)

In order to elucidate the mechanisms underlying resistance to the PARG inhibitor PDD00017273 (Figure 1A), a PDD00017273‐resistant variant of the HCT116 human colorectal cancer cell line was developed. PDD00017273 is the first cell‐active PARG inhibitor.^11^ HCT116R^PDD^ cells resistant to PDD00017273 were generated by repeatedly exposing parental HCT116 cells to escalating concentrations of PDD00017273 for ~4 weeks at 10 μM and 4 weeks at 30 μM, respectively (Figure 1B). The HCT116R^PDD^ and parental HCT116 cells exhibited nearly identical morphological characteristics (Figure 1C). After 72 h of continuous exposure, the EC_50_ of PDD00017273 in HCT116R^PDD^ and parental HCT116 cells was determined using the WST‐8 assay. As shown in Figure 1D and Table 1, the EC_50_ value of HCT116R^PDD^ cells was greater (>100 μM) than that of HCT116 parental cells (EC_50_ = 43.7 ± 13.0 μM). At 100 μM PDD00017273, the cell activity of CRC cells was ~31% in HCT116 cells and 75% in HCT116R^PDD^ cells. After 10 days of continuous exposure, the EC_50_ of PDD00017273 in HCT116R^PDD^ and parental HCT116 cells was determined using a colony formation assay. As shown in Figure 1E and Table 1, HCT116R^PDD^ cells were significantly more resistant to the PARG inhibitor PDD00017273 than their parental HCT116 counterparts (EC_50_ = 60.0 ± 17.6 μM). The colony formation of HCT116R^PDD^ and HCT116 cells treated with 100 μM PDD00017273 was evaluated, and that of HCT116R^PDD^ was significantly greater than that of the sensitive parental HCT116 cells. Notably, HCT116R^PDD^ cell colonies were smaller than the HCT116 parent cell colonies (Figure 1E). Moreover, treatment of HCT116R^PDD^ cells with 100 μM PDD00017273 results in a maximum of 8%–10% cell death at each time point (Figure 1F). Treatment of HCT116 cells with 100 μM PDD00017273 induces time‐dependent cell death (up to 19% in 72 h) (Figure 1F). According to these results, HCT116R^PDD^ cells are resistant to the selective PARG inhibitor PDD00017273.

**TABLE 1:** Anticancer sensitivities to PARG inhibitors PDD00017273 and COH34 of the parental HCT116 and HCT116R^PDD^ cells

PARG inhibitor	EC_50_ (μM)		Assay
	HCT116	HCT116R^PDD^	
PDD00017273	43.7 ± 13.0	N.D.	WST‐8
	60.0 ± 17.6	N.D.	Colony formation
COH34	8.3 ± 2.8	8.2 ± 2.2	WST‐8
	6.1 ± 0.9	5.3 ± 0.1	Colony formation

*Note*: EC_50_ values indicate mean ± SD.

Abbreviation: N.D., not detected.

### 3.2 Analysis of PARG, PARPs, and related gene mutations by exome sequencing

Single nucleotide variants (SNVs), insertions (INSs), and deletions (DELs) were identified as genetically distinct gene variants and alterations in parental HCT116 cells and PDD00017273‐resistant HCT116R^PDD^ cells using whole‐exome sequencing (Table 2). In parental HCT116 cells and PDD00017273‐resistant HCT116R^PDD^ cells, the genetic variants of PARylation and related reactions, such as *PARG* and *PARP1*, were analyzed (Tables 3 and S1). Almost all PARP family and related gene mutation profiles were identical in both cells (Table S1). The genetic mutation profiles of *PARG*, *PARP1*, *PARP8*, *PARP10*, *BRCA1*, and *ZC3HAV1* were notably distinct between HCT116 and HCT116R^PDD^ cells. In the HCT116R^PDD^ cells, one functional *PARG* variant, 1054G > C (Glu352Gln), was identified. Moreover, HCT116R^PDD^ cells exhibited the *PARP1* variant 402G > T (Lys134Asn).

**TABLE 2:** Number of genetically different gene variants compared to HCT116 cells and HCT116R^PDD^ cells

Variant type		HCT116	HCT116R^PDD^
SNV		109 170	104 799
	Synonymous variant	12 967	13 160
	Missense variant	14 133	14 040
	Stop gained	247	237
	Stop lost	34	36
INDEL		36 954	32 029
	Frameshift variant	1381	1341
	Inframe insertion	183	186
	Inframe deletion	284	281

Abbreviations: INDEL, insertion/deletion; SNV, single nucleotide variants.

**TABLE 3:** Gene mutation of *PARG*, *PARP* family, and related genes in the parental HCT116 and HCT116R^PDD^ cells

Gene name	HCT116	HCT116R^PDD^
*PARG*	wt	mt(Glu352Gln)het
*PARP1*	wt	mt(Lys134Asn)het
*PARP8*	wt	mt(Ala548Val)het
*PARP10*	mt(Val682Leu)het	wt
*ZC3HAV1*	wt	mt(Tyr814Tyr)het
*BRCA1*	mt(Lys739Asn)het	wt

Abbreviations: het, heterozygous; hom, homozygous; mt, mutation‐type; wt, wild‐type.

### 3.3 Regulation of PARG, PARP, and intracellular PARylation in the parental HCT116 cells and PARG inhibitor‐resistant HCT116R^PDD^ cells

**FIGURE 2:**
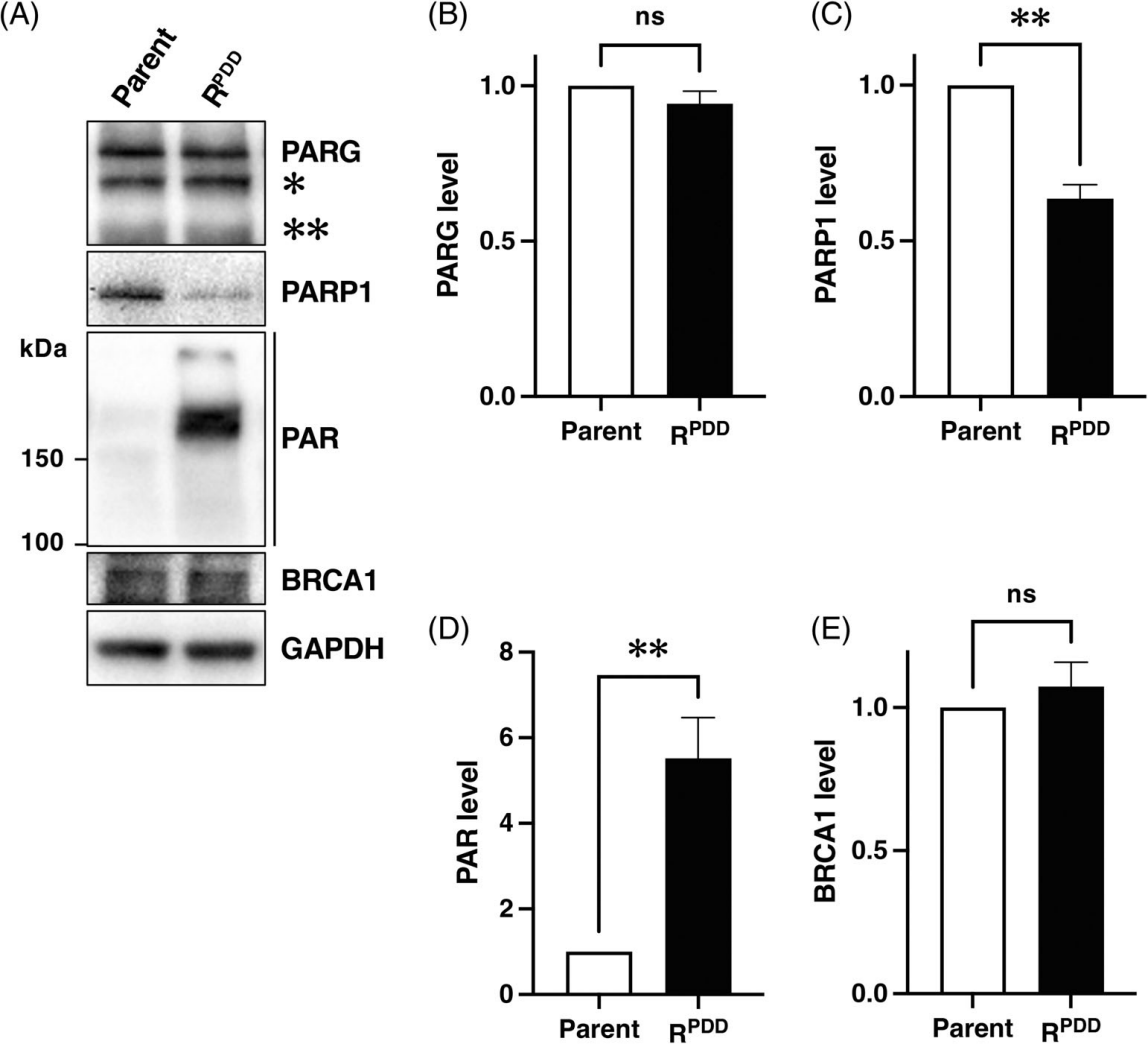
Characteristics of PARG, PARP1 protein, and intracellular PAR levels in the PDD00017273‐resistant HCT116R^PDD^ and parental HCT116 cells. (A) Under untreated conditions, whole‐cell lysates were prepared from parental HCT116 and HCT116R^PDD^ cells (without PDD00017273). The levels of PARG, PARP1, PAR, BRCA1, and GAPDH were measured using western blot analysis. * indicates 60 kDa PARG isoform. ** indicate 55 kDa PARG isoform. The GAPDH gene was utilized as an internal control. There were at least three independent experiments represented in the data. Levels of (B) PARG, (C) PARP1, (D) PAR, and (E) BRCA1, as well as the parental HCT116 and HCT116R^PDD^ cells. PARG, PARP1, PAR, and BRCA1 levels in HCT116R^PDD^ cells were represented by the ratio of PARG, PARP1, PAR, or BRCA1 density to GAPDH density, relative to the value of parental HCT116 cells. The results are the averages of three independent experiments, with error bars showing ±SE of triplicate. ns, no significant difference. **p < .01

In order to investigate the regulation of PARG, PARP, and PARylation by PDD00017273 resistance, western blot analysis was used to examine the protein levels of PARG, PARP, BRCA1, and intracellular PAR in parental HCT116 and PDD00017273‐resistant HCT116R^PDD^ cells (Figure 2). Figure 2A (upper panel) and 2B demonstrate that the PARG protein levels in both cells were nearly identical. Interestingly, PARP1 protein levels in HCT116R^PDD^ cells were 0.7‐fold lower than in HCT116 cells (Figure 2A, second panel, and 2C). Additionally, intracellular PAR levels in HCT116R^PDD^ cells were 5.5‐fold higher than in HCT116 parental cells (Figure 2A, third panel, and 2D). Furthermore, the BRCA1 protein levels in HCT116 and HCT116R^PDD^ cells were nearly identical (Figure 2A, fourth panel, and 2E). The GAPDH was employed as an internal control (Figure 2A, bottom panel).

### 3.4 Drug sensitivity of PDD00017273‐resistant HCT116 cells to another chemically and structurally different PARG inhibitor, COH34

**FIGURE 3:**
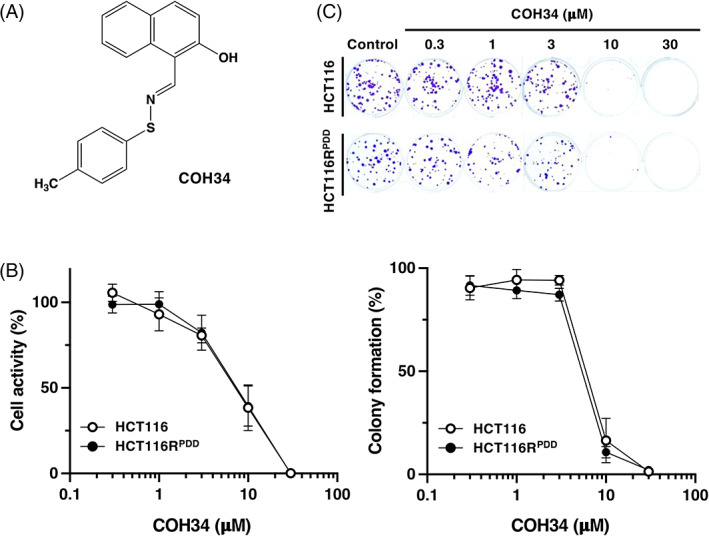
The sensitivity of the PDD00017273‐resistant HCT116R^PDD^ and parental HCT116 cells to another PARG inhibitor, COH34. (A) The chemical structure of COH34, a PARG inhibitor. (B) After 72 h of treatment with COH34, the cellular activity of HCT116 and HCT116R^PDD^ cells was evaluated. The results were the average of three independent samples, with error bars showing ±SE in triplicate. The white circle represents HCT116 cells; black circle represents HCT116R^PDD^ cells. (C) Image of colony formation assay on 6‐well plate. After 10 days of treatment with the indicated concentrations of COH34, both HCT116R^PDD^ and parental HCT116 cells form colonies. Colony formation (%) represents the averages of three independent experiments, and each conducted in duplicate, with error bars showing mean ± SE of triplicate values. White circle, HCT116 cells; black circle, HCT116R^PDD^ cells

The effect of another chemically and structurally distinct PARG inhibitor, COH34,^12^ on the proliferation of parental HCT116 and PDD00017273‐resistant HCT116R^PDD^ cells was investigated using the WST‐8 assay and colony formation (Figure 3). The highly potent and selective PARG inhibitor, COH34, is a recently reported small molecule compound,^12^ as depicted in Figure 3A. In addition, as shown in Figure 3B and Table 1, HCT116R^PDD^ cells were as sensitive to the novel PARG inhibitor COH34 as the parental HCT116 cells in the cell activity WST‐8 assay (EC_50_ = 8.2 ± 2.2 μM in HCT116R^PDD^ cells; EC_50_ = 8.3 ± 2.8 μM in HCT116). Similar results were obtained by colony formation assay (EC_50_ = 5.3 ± 0.1 μM in HCT116R^PDD^ cells and EC_50_ = 6.1 ± 0.9 μM in HCT116 cells) (Figure 3C and Table 1). These results indicate that PDD00017273‐resistant HCT116R^PDD^ cells do not express COH34 crossresistance.

## 4 DISCUSSION

Cancer cells can acquire resistance to anticancer drugs through various mechanisms, including drug target alteration, drug inactivation, drug efflux, bypass pathway activation, DNA repair activation, and cell death escape.^30^, ^31^ PARG is a crucial enzyme in the PAR metabolic pathway and a desirable anticancer drug target.^1^, ^2^, ^3^, ^4^ The PARG inhibitor PDD00017273 provides beneficial fundamental insights into the function of PARG and its significance as a molecular target for cancer treatment.^5^ The PDD00017273‐resistant HCT116R^PDD^ cells were established, and the biological aspects and resistance mechanisms were investigated. Interestingly, in HCT116R^PDD^ cells, a single functional *PARG* variant, 1054G > C (Glu352Gln), was identified. Furthermore, HCT116R^PDD^ cells contained the *PARP1* variant 402G > T (Lys134Asn). Notably, the *PARG* variant 1054G > C (Glu352Gln) resides in the region encoding the protein's putative regulatory domain.^32^ Similarly, the identified *PARP1* variant, 402G > T (Lys134Asn), resides in the zinc finger II region within the DNA‐binding domain of *PARP1*.^33^, ^34^ Three zinc fingers were located at the N‐terminus of the DNA‐binding domain of *PARP1*: zinc finger I, zinc finger II, and zinc finger III.^35^ The absence of the zinc finger I and II regions significantly reduces PARP1's DNA‐binding affinity and the loss of enzymatic activity of *PARP1*.^33^, ^36^ These findings indicate that the mutated *PARG* protein in HCT116R^PDD^ cells has acquired resistance to PDD00017273 as a result of structural alterations. Similarly, *PARP1* mutations in HCT116R^PDD^ cells may influence PARP1 activity and function. In addition, these results indicate that PDD00017273 resistance induces gene mutations in *PARP1*, *PARP8*, and *ZC3HAV*. Furthermore, it was discovered that HCT116R^PDD^ cells changed the *BRCA1* gene mutation 2217A > C (Lys739Asn) in parental HCT116 cells to the wild ‐type sequence. Previous studies have examined the correlation between *BRCA1/2* mutation and PARG inhibitor sensitivity in numerous cancer cells.^11^, ^12^ In our study, we found no association between the *BRCA1* mutation and resistance to the PARG inhibitor PDD00017273 and sensitivity to the PARG inhibitors PDD00017273 and COH34. Importantly, it is required to investigate the frequency and reproducibility of biological and genetic characteristics in HCT116 cells resistant to PDD00017273. We recognize that additional studies in multiple PDD00017273‐resistant human cancer cells using identified gene mutations such as *PARG* and *PARP1* are required to comprehend the mechanisms of PDD00017273 resistance.

Moreover, these findings suggest that the Glu352Gln PARG mutation primarily affects PARG conformation and its activity or function. In the future, we recognize the need to further elucidate the relationship between the PARG inhibitor resistance mechanisms and the identified *PARG* and *PARP1* variants through detailed genetic analysis by targeted sequencing and biochemical analysis using the recombinant PARG and PARP1 variant proteins. Most importantly, the overall structure of the PARG protein remains unknown. Furthermore, according to our findings, resistance to the PARG inhibitor PDD00017273 induces the *PARP1* mutation in Lys134Asn and significantly reduces *PARP1* expression. Interestingly, both HCT116 and HCT116R^PDD^ cells were sensitive to COH34, a chemically and structurally distinct PARG inhibitor. This finding indicates that the Glu352Gln mutation in PARG may impact drug sensitivity to PDD00017273 but not to COH34. Our novel findings may indicate that the mutated *PARG* has acquired resistance to PDD00017273 because of structural modifications. Moreover, these findings indicate that resistance to PDD00017273 induces mutation and *PARP* downregulation.

In conclusion, our findings suggest that resistance to the selective PARG inhibitor PDD00017273 induces *PARG* mutation and *PARP* downregulation in human colorectal cancer HCT116 cells. Importantly, our findings improve comprehension of the PARG‐targeted anticancer inhibitor in terms of resistance mechanisms and anticancer chemotherapeutic strategies.

## AUTHOR CONTRIBUTIONS

**Kaede Tsuda:** Data curation (equal); formal analysis (equal); investigation (lead); writing – review and editing (equal). **Chinatsu Kurasaka:** Formal analysis (equal); investigation (supporting); methodology (equal); writing – review and editing (equal). **Yoko Ogino:** Data curation (equal); investigation (supporting); methodology (equal); writing – review and editing (equal). **Akira Sato:** Conceptualization (lead); data curation (lead); formal analysis (lead); investigation (supporting); methodology (lead); project administration (lead); supervision (lead); validation (lead); visualization (lead); writing – original draft (lead); writing – review and editing (lead).

## CONFLICT OF INTEREST

The authors have stated explicitly that there are no conflicts of interest in connection with this article.

## ETHICS STATEMENT

Not applicable.

## Supporting information


**TABLE S1** Gene mutation status of *PARG*, *PARP* family, and related genes in the parental HCT116 and HCT116R^PDD^ cells.

## Data Availability

The data that support the findings of this study are available from the corresponding author upon reasonable request.

## References

[1] Masutani M , Nakagama H , Sugimura T . Poly(ADP‐ribose) and carcinogenesis. Genes Chromosomes Cancer. 2003;38(4):339‐348. doi:10.1002/gcc.10250 14566854

[2] Miwa M , Masutani M . PolyADP‐ribosylation and cancer. Cancer Sci. 2007;98(10):1528‐1535. doi:10.1111/j.1349-7006.2007.00567.x 17645773 PMC11159171

[3] Wang J , Sato A , Fujimori H , Miki Y , Masutani M . PARP and carcinogenesis. In: Curtin NJ , Sharma RA , eds. PARP Inhibitors for Cancer Therapy. Springer International Publishing; 2015:99‐124.

[4] Tanuma S , Sato A , Oyama T , Yoshimori A , Abe H , Uchiumi F . New insights into the roles of NAD+‐poly(ADP‐ribose) metabolism and poly(ADP‐ribose) glycohydrolase. Curr Protein Pept Sci. 2016;17(7):668‐682. doi:10.2174/1389203717666160419150014 27817743

[5] Slade D . PARP and PARG inhibitors in cancer treatment. Genes Dev. 2020;34(5–6):360‐394. doi:10.1101/gad.334516.119 32029455 PMC7050487

[6] Miwa M , Tanaka M , Matsushima T , Sugimura T . Purification and properties of a glycohydrolase from calf thymus splitting ribose‐ribose linkages of poly(adenosine diphosphate ribose). J Biol Chem. 1974;249(11):3475‐3482. doi:10.1016/S0021-9258(19)42597-0 4831224

[7] Oka S , Kato J , Moss J . Identification and characterization of a mammalian 39‐kDa poly(ADP‐ribose) glycohydrolase*. J Biol Chem. 2006;281(2):705‐713. doi:10.1074/jbc.M510290200 16278211

[8] Menear KA , Adcock C , Boulter R , et al. 4‐[3‐(4‐cyclopropanecarbonylpiperazine‐1‐carbonyl)‐4‐fluorobenzyl]‐2H‐phthalazin‐1‐one: a novel bioavailable inhibitor of poly(ADP‐ribose) polymerase‐1. J Med Chem. 2008;51(20):6581‐6591. doi:10.1021/jm8001263 18800822

[9] Islam R , Koizumi F , Kodera Y , Inoue K , Okawara T , Masutani M . Design and synthesis of phenolic hydrazide hydrazones as potent poly(ADP‐ribose) glycohydrolase (PARG) inhibitors. Bioorg Med Chem Lett. 2014;24(16):3802‐3806. doi:10.1016/j.bmcl.2014.06.065 25042255

[10] Sasaki Y , Hozumi M , Fujimori H , et al. PARG inhibitors and functional PARG inhibition models. Curr Protein Pept Sci. 2016;17(7):641‐653. doi:10.2174/1389203717666160419145130 27817742

[11] James DI , Smith KM , Jordan AM , et al. First‐in‐class chemical probes against poly(ADP‐ribose) glycohydrolase (PARG) inhibit DNA repair with differential pharmacology to olaparib. ACS Chem Biol. 2016;11(11):3179‐3190. doi:10.1021/acschembio.6b00609 27689388

[12] Chen SH , Yu X . Targeting dePARylation selectively suppresses DNA repair‐defective and PARP inhibitor‐resistant malignancies. Sci Adv. 2019;5(4):eaav4340. doi:10.1126/sciadv.aav4340 30989114 PMC6457938

[13] Fathers C , Drayton RM , Solovieva S , Bryant HE . Inhibition of poly(ADP‐ribose) glycohydrolase (PARG) specifically kills BRCA2‐deficient tumor cells. Cell Cycle. 2012;11(5):990‐997. doi:10.4161/cc.11.5.19482 22333589

[14] Gravells P , Grant E , Smith KM , James DI , Bryant HE . Specific killing of DNA damage‐response deficient cells with inhibitors of poly(ADP‐ribose) glycohydrolase. DNA Repair (Amst). 2017;52:81‐91. doi:10.1016/j.dnarep.2017.02.010 28254358 PMC5360195

[15] Gravells P , Neale J , Grant E , et al. Radiosensitization with an inhibitor of poly(ADP‐ribose) glycohydrolase: a comparison with the PARP1/2/3 inhibitor olaparib. DNA Repair (Amst). 2018;61:25‐36. doi:10.1016/j.dnarep.2017.11.004 29179156 PMC5765821

[16] Marques M , Jangal M , Wang LC , et al. Oncogenic activity of poly (ADP‐ribose) glycohydrolase. Oncogene. 2019;38(12):2177‐2191. doi:10.1038/s41388-018-0568-6 30459355 PMC6484711

[17] Shirai H , Poetsch AR , Gunji A , et al. PARG dysfunction enhances DNA double strand break formation in S‐phase after alkylation DNA damage and augments different cell death pathways. Cell Death Dis. 2013;4(6):e656. doi:10.1038/cddis.2013.133 23744356 PMC3698538

[18] Wei L , Nakajima S , Hsieh CL , et al. Damage response of XRCC1 at sites of DNA single strand breaks is regulated by phosphorylation and ubiquitylation after degradation of poly(ADP‐ribose). J Cell Sci. 2013;126(Pt 19):4414‐4423. doi:10.1242/jcs.128272 23868975 PMC3784821

[19] Chand SN , Zarei M , Schiewer MJ , et al. Posttranscriptional regulation of PARG mRNA by HuR facilitates DNA repair and resistance to PARP inhibitors. Cancer Res. 2017;77(18):5011‐5025. doi:10.1158/0008-5472.CAN-16-2704 28687616 PMC5663502

[20] Jain A , Agostini LC , McCarthy GA , et al. Poly (ADP) ribose glycohydrolase can be effectively targeted in pancreatic cancer. Cancer Res. 2019;79(17):4491‐4502. doi:10.1158/0008-5472.CAN-18-3645 31273064 PMC6816506

[21] Pillay N , Tighe A , Nelson L , et al. DNA replication vulnerabilities render ovarian cancer cells sensitive to poly(ADP‐ribose) glycohydrolase inhibitors. Cancer Cell. 2019;35(3):519‐33.e8. doi:10.1016/j.ccell.2019.02.004 30889383 PMC6428690

[22] Sasaki Y , Fujimori H , Hozumi M , et al. Dysfunction of poly (ADP‐ribose) glycohydrolase induces a synthetic lethal effect in dual specificity phosphatase 22‐deficient lung cancer cells. Cancer Res. 2019;79(15):3851‐3861. doi:10.1158/0008-5472.CAN-18-1037 31142510

[23] Ali R , Alblihy A , Miligy IM , et al. Molecular disruption of DNA polymerase β for platinum sensitisation and synthetic lethality in epithelial ovarian cancers. Oncogene. 2021;40(14):2496‐2508. doi:10.1038/s41388-021-01710-y 33674744 PMC8032555

[24] Noordermeer SM , van Attikum H . PARP inhibitor resistance: a tug‐of‐war in BRCA‐mutated cells. Trends Cell Biol. 2019;29(10):820‐834. doi:10.1016/j.tcb.2019.07.008 31421928

[25] Li H , Liu Z‐Y , Wu N , Chen Y‐C , Cheng Q , Wang J . PARP inhibitor resistance: the underlying mechanisms and clinical implications. Mol Cancer. 2020;19(1):107. doi:10.1186/s12943-020-01227-0 32563252 PMC7305609

[26] Ogino Y , Sato A , Uchiumi F , Tanuma SI . Cross resistance to diverse anticancer nicotinamide phosphoribosyltransferase inhibitors induced by FK866 treatment. Oncotarget. 2018;9(23):16451‐16461. doi:10.18632/oncotarget.24731 29662658 PMC5893253

[27] Kurasaka C , Ogino Y , Sato A . Molecular mechanisms and tumor biological aspects of 5‐fluorouracil resistance in HCT116 human colorectal cancer cells. Int J Mol Sci. 2021;22(6):2916. doi:10.3390/ijms22062916 33805673 PMC8002131

[28] Ogino Y , Sato A , Uchiumi F , Tanuma SI . Genomic and tumor biological aspects of the anticancer nicotinamide phosphoribosyltransferase inhibitor FK866 in resistant human colorectal cancer cells. Genomics. 2019;111(6):1889‐1895. doi:10.1016/j.ygeno.2018.12.012 30582964

[29] Sato A , Satake A , Hiramoto A , Wataya Y , Kim HS . Protein expression profiles of necrosis and apoptosis induced by 5‐fluoro‐2′‐deoxyuridine in mouse cancer cells. J Proteome Res. 2010;9(5):2329‐2338. doi:10.1021/pr9010537 20155980

[30] Longley DB , Harkin DP , Johnston PG . 5‐fluorouracil: mechanisms of action and clinical strategies. Nat Rev Cancer. 2003;3(5):330‐338. doi:10.1038/nrc1074 12724731

[31] Blondy S , David V , Verdier M , Mathonnet M , Perraud A , Christou N . 5‐fluorouracil resistance mechanisms in colorectal cancer: from classical pathways to promising processes. Cancer Sci. 2020;111(9):3142‐3154. doi:10.1111/cas.14532 32536012 PMC7469786

[32] Mortusewicz O , Fouquerel E , Amé JC , Leonhardt H , Schreiber V . PARG is recruited to DNA damage sites through poly(ADP‐ribose)‐ and PCNA‐dependent mechanisms. Nucleic Acids Res. 2011;39(12):5045‐5056. doi:10.1093/nar/gkr099 21398629 PMC3130271

[33] Langelier MF , Planck JL , Roy S , Pascal JM . Crystal structures of poly(ADP‐ribose) polymerase‐1 (PARP‐1) zinc fingers bound to DNA: structural and functional insights into DNA‐dependent PARP‐1 activity. J Biol Chem. 2011;286(12):10690‐10701. doi:10.1074/jbc.M110.202507 21233213 PMC3060520

[34] Kamaletdinova T , Fanaei‐Kahrani Z , Wang ZQ . The enigmatic function of PARP1: from PARylation activity to PAR readers. Cells. 2019;8(12):1625. doi:10.3390/cells8121625 31842403 PMC6953017

[35] Buelow B , Uzunparmak B , Paddock M , Scharenberg AM . Structure/function analysis of PARP‐1 in oxidative and nitrosative stress‐induced monomeric ADPR formation. PLoS One. 2009;4(7):e6339. doi:10.1371/journal.pone.0006339 19641624 PMC2713433

[36] Smulson M , Istock N , Ding R , Cherney B . Deletion mutants of poly(ADP‐ribose) polymerase support a model of cyclic association and dissociation of enzyme from DNA ends during DNA repair. Biochemistry. 1994;33(20):6186‐6191. doi:10.1021/bi00186a018 8193132

