# Supplementary material for: Genomic and biological aspects of resistance to selective poly(ADP‐ribose) glycohydrolase inhibitor PDD00017273 in human colorectal cancer cells
Source: Cancer Rep (Hoboken). 2022 Aug 26;6(2):e1709. doi: 10.1002/cnr2.1709 (PMC9939995; doi:10.1002/cnr2.1709)
Supplement: Supplementary file 1 — TABLE S1 Gene mutation status of PARG, PARP family, and related genes in the parental HCT116 and HCT116RPDD cells. [file CNR2-6-e1709-s001.pdf]

Supplementary Table 1. Gene mutation status of *PARG*, *PARP* family, and related genes in the parental HCT116 and HCT116R^{PDD} cells

**Gene name**	**HCT116**	**HCT116R^{PDD}**	**HCT116**	**HCT116R^{PDD}**	**chromosome localization**
	**Nucleotide information**		**Amino acid information**		
PARG	**wt**	**1054G>C**	**wt**	**mt(Glu352Gln)het**	chr10
PARP1	2285T>C	2285T>C	mt(Val762Ala)het	mt(Val762Ala)het	chr1
	1056A>G	1056A>G	mt(Lys352Lys)het	mt(Lys352Lys)het	chr1
	852T>C	852T>C	mt(Ala284Ala)het	mt(Ala284Ala)het	chr1
	243C>T	243C>T	mt(Asp81Asp)het	mt(Asp81Asp)het	chr1
	**wt**	**402G>T**	**wt**	**mt(Lys134Asn)het**	chr1
PARP2	1131T>C	1131T>C	mt(Tyr377Tyr)hom	mt(Tyr377Tyr)hom	chr14
PARP3	wt	wt	wt	wt	chr3
PARP4	4966G>C	4966G>C	mt(Ala1656Pro)hom	mt(Ala1656Pro)hom	chr13
	4691T>C	4691T>C	mt(Ile1564Thr)hom	mt(Ile1564Thr)hom	chr13
	4649T>C	4649T>C	mt(Leu1550Pro)hom	mt(Leu1550Pro)hom	chr13
	4376C>A	4376C>A	mt(Ser1459Tyr)hom	mt(Ser1459Tyr)hom	chr13
	4180T>G	4180T>G	mt(Ser1394Ala)hom	mt(Ser1394Ala)hom	chr13
	3982C>A	3982C>A	mt(Pro1328Thr)hom	mt(Pro1328Thr)hom	chr13
	3838G>T	3838G>T	mt(Gly1280Cys)het	mt(Gly1280Cys)het	chr13
	3838G>C	3838G>C	mt(Gly1280Arg)het	mt(Gly1280Arg)het	chr13
	3794G>C	3794G>C	mt(Gly1265Ala)hom	mt(Gly1265Ala)hom	chr13
	3642G>A	3642G>A	mt(Glu1214Glu)hom	mt(Glu1214Glu)hom	chr13
	3558G>A	3558G>A	mt(Ser1186Ser)hom	mt(Ser1186Ser)hom	chr13
	3322C>T	3322C>T	mt(Arg1108Cys)hom	mt(Arg1108Cys)hom	chr13
	3239T>G	3239T>G	mt(Leu1080Arg)hom	mt(Leu1080Arg)hom	chr13
	3228G>A	3228G>A	mt(Pro1076Pro)het	mt(Pro1076Pro)het	chr13
	3194T>C	3194T>C	mt(Val1065Ala)het	mt(Val1065Ala)het	chr13
	3176A>G	3176A>G	mt(Gln1059Arg)het	mt(Gln1059Arg)het	chr13
	3116T>C	3116T>C	mt(Ile1039Thr)het	mt(Ile1039Thr)het	chr13
	2807T>C	2807T>C	mt(Met936Thr)hom	mt(Met936Thr)hom	chr13
	2695G>A	2695G>A	mt(Ala899Thr)hom	mt(Ala899Thr)hom	chr13
PARP6	1348C>T	1348C>T	mt(Leu450Leu)het	mt(Leu450Leu)het	chr15
PARP8	420G>A	420G>A	mt(Gly140Gly)het	mt(Gly140Gly)het	chr5
	1527T>C	1527T>C	mt(Cys509Cys)het	mt(Cys509Cys)het	chr5
	**wt**	**1643C>T**	**wt**	**mt(Ala548Val)het**	chr5
PARP9	1583A>G	1583A>G	mt(Tyr528Cys)het	mt(Tyr528Cys)het	chr3
	366C>G	366C>G	mt(Val122Val)het	mt(Val122Val)het	chr3

PARP10	**2044G>T**	**wt**	**mt(Val682Leu)het**	**wt**	chr8
PARP11	wt	wt	wt	wt	chr12
PARP12	1911G>A	1911G>A	mt(Pro637Pro)het	mt(Pro637Pro)het	chr7
	1569C>T	1569C>T	mt(Phe523Phe)het	mt(Phe523Phe)het	chr7
	449G>T	449G>T	mt(Trp150Leu)het	mt(Trp150Leu)het	chr7
PARP14	3965delA	3965delA	mt(Asn1322fs)het	mt(Asn1322fs)het	chr3
	4323T>C	4323T>C	mt(Tyr1441Tyr)hom	mt(Tyr1441Tyr)hom	chr3
PARP15	1743A>G	1743A>G	mt(Lys581Lys)het	mt(Lys581Lys)het	chr3
	1882G>A	1882G>A	mt(Gly628Arg)het	mt(Gly628Arg)het	chr3
PARP16	wt	wt	wt	wt	chr15
TNKS	1386A>G	1386A>G	mt(Thr462Thr)hom	mt(Thr462Thr)hom	chr8
	1806T>C	1806T>C	mt(Gly602Gly)hom	mt(Gly602Gly)hom	chr8
	2200G>A	2200G>A	mt(Glu734Lys)het	mt(Glu734Lys)het	chr8
	3846G>A	3846G>A	mt(Pro1282Pro)het	mt(Pro1282Pro)het	chr8
TNKS2	1865dupA	1865dupA	mt(Asn622fs)het	mt(Asn622fs)het	chr10
	3431_3433delGAG	3431_3433delGAG	mt(Gly1144del)het	mt(Gly1144del)het	chr10
TIPARP	wt	wt	wt	wt	chr3
ZC3HAV1	**wt**	**2442C>T**	**wt**	**mt(Tyr814Tyr)het**	chr7
TP53	215C>G	215C>G	mt(Pro72Arg)hom	mt(Pro72Arg)hom	chr17
ADPRHL2	wt	wt	wt	wt	chr1
BRCA1	**2217A>C**	**wt**	**mt(Lys739Asn)het**	**wt**	chr17
BRCA2	3396A>G	3396A>G	mt(Lys1132Lys)het	mt(Lys1132Lys)het	chr13
	3807T>C	3807T>C	mt(Val1269Val)het	mt(Val1269Val)het	chr13
	4563A>G	4563A>G	mt(Leu1521Leu)hom	mt(Leu1521Leu)hom	chr13
	6513G>C	6513G>C	mt(Val2171Val)hom	mt(Val2171Val)hom	chr13
	7242A>G	7242A>G	mt(Ser2414Ser)het	mt(Ser2414Ser)het	chr13
	7397T>C	7397T>C	mt(Val2466Ala)hom	mt(Val2466Ala)hom	chr13
	8021dupA	8021dupA	mt(Ile2675fs)het	mt(Ile2675fs)het	chr13
PALB2	wt	wt	wt	wt	chr16
FAM175A	wt	wt	wt	wt	chr4
BARD1	1134G>C	1134G>C	mt(Arg378Ser)het	mt(Arg378Ser)het	chr2
	1075_1095delTTGCCTGAATGTTCTTCACCA	1075_1095delTTGCCTGAATGTTCTTCACCA	mt(Leu359_Pro365del)het	mt(Leu359_Pro365del)het	chr2
TIMELESS	2492G>A	2492G>A	mt(Arg831Gln)hom	mt(Arg831Gln)hom	chr12

	1363A>T	1363A>T	mt(Ile455Leu)hom	mt(Ile455Leu)hom	chr12
	765G>A	765G>A	mt(Val255Val)hom	mt(Val255Val)hom	chr12
HUS1	wt	wt	wt	wt	chr7
RFC2	312A>G	312A>G	mt(Glu104Glu)het	mt(Glu104Glu)het	chr7
DUSP22	114C>T	114C>T	mt(His38His)het	mt(His38His)het	chr6
	573G>A	573G>A	mt(Pro191Pro)het	mt(Pro191Pro)het	chr6
POLB	725C>G	725C>G	mt(Pro242Arg)het	mt(Pro242Arg)het	chr8

Note. wt, wild-type; mutation-type; hom, homozygous; het, heterozygous.
